# Supplementary material for: Chromothripsis during telomere crisis is independent of NHEJ, and consistent with a replicative origin
Source: Genome Res. 2019 May;29(5):737–49. doi: 10.1101/gr.240705.118 (PMC6499312; doi:10.1101/gr.240705.118)
Supplement: Supplemental Material [file supp_gr.240705.118_Supplemental_file_1.zip › contigs/annotated_contigs/DB53/contig.2.DB53_length_595_mean_cov_3.85378151261.docx]

**DB53_length_595_mean_cov_3.85378151261**

```
CATTATGTCATTTTGTGTTGTCAGCAGCCATTTCTGATCAGTGGTTAGATATCTAATTAATTAGAGATTGAAAAGGATGATATTCCAGT
 >chr8:133718759-133719139 - E=5e-217
TTGATCACATCTTCTTTATTGGGAATATATCTGTGCAAGGAAACATCTACCGTCTGATTGCCCAGTGGTTCAGATTTTATAGGAAAGGA

GAGATGAATGCTTGAGTCTTTTCCTGTATTTGCTAATTTTCAAAACAAAAAGTTTATTTCTTAATATCTTTTAAAGGTGGCCAGGATGC

TATGTATAATCAGCATTTATATATATGAAGGAACCATGTTTAATATATAAAATTTCCCAAAAGGCACACCCTCCAACTTCCAAACTCTA

TATACTTTGTACCTCATCAAGATG|GATAA|TATTGAGAGTTGCTTTATCACCAAGCATGTGGGCAATCTTAGAGTATGTTCCATATGC
                              >chr8:130825355-130825565 + E=1e-114
AGATGAGAAGAATATATAATCTGGGTAGCTGGGTGGAATATTCTGTAGATTTCTATTAGGTCCAATTAGTCAAGTGTCAAATTTAAGTC

CAGAATTTGTTAGTTTCCTGCCTCAATAATCTAATGCTGTCAGGGGAGTGTTGAAGTCCCCCA
```
